# Tight binding of cytochrome *b*_5_ to cytochrome P450 17A1 is a critical feature of stimulation of C21 steroid lyase activity and androgen synthesis

**DOI:** 10.1016/j.jbc.2021.100571

**Published:** 2021-03-20

**Authors:** Donghak Kim, Vitchan Kim, Kevin D. McCarty, F. Peter Guengerich

**Affiliations:** 1 Department of Biochemistry, Vanderbilt University School of Medicine, Nashville, Tennessee, USA; 2 Department of Biological Sciences, Konkuk University, Seoul, Republic of Korea

**Keywords:** cytochrome P450, cytochrome *b*_5_, enzyme kinetics, enzyme mechanism, enzyme inhibitor, enzyme catalysis, fluorescence, *b*_5_, cytochrome *b*_5_, 17α-OH, 17α-hydroxy, NTA, nitrilotriacetate, P450, cytochrome P450, POR, NADPH–P450 reductase, SPR, surface plasmon resonance

## Abstract

It has been recognized for >50 years that cytochrome *b*_5_ (*b*_5_) stimulates some cytochrome P450 (P450)–catalyzed oxidations, but the basis of this function is still not understood well. The strongest stimulation of catalytic activity by *b*_5_ is in the P450 17A1 lyase reaction, an essential step in androgen synthesis from 21-carbon (C21) steroids, making this an excellent model system to interrogate *b*_5_ function. One of the issues in studying *b*_5_–P450 interactions has been the limited solution assay methods. We constructed a fluorescently labeled variant of human *b*_5_ that can be used in titrations. The labeled *b*_5_ bound to WT P450 17A1 with a *K*_*d*_ of 2.5 nM and rapid kinetics, on the order of 1 s^−1^. Only weak binding was observed with the clinical P450 17A1 variants E305G, R347H, and R358Q; these mutants are deficient in lyase activity, which has been hypothesized to be due to attenuated *b*_5_ binding. *K*_*d*_ values were not affected by the presence of P450 17A1 substrates. A peptide containing the P450 17A1 Arg-347/Arg-358 region attenuated Alexa 488-T70C-*b*_5_ fluorescence at higher concentrations. The addition of NADPH–P450 reductase (POR) to an Alexa 488-T70C-*b*_5_:P450 17A1 complex resulted in a concentration-dependent partial restoration of *b*_5_ fluorescence, indicative of a ternary P450:*b*_5_:POR complex, which was also supported by gel filtration experiments. Overall, these results are interpreted in the context of a dynamic and tight P450 17A1:*b*_5_ complex that also binds POR to form a catalytically competent ternary complex, and variants that disrupt this interaction have low catalytic activity.

Cytochrome P450 (P450, CYP) enzymes are the major catalysts involved in the oxidation of chemicals (1). In addition to playing major roles in areas as diverse as drug metabolism and the biosynthesis of natural products, P450s are the major catalysts involved in steroid metabolism and catalyze most of the oxidations beginning with the side-chain cleavage of cholesterol to form pregnenolone (2). P450 17A1 plays a critical role in the synthesis of androgens as well as in the generation of 17α-hydroxy (17α-OH) steroids and in the synthesis of mineralocorticoids and glucocorticoids (Fig. 1). More than 125 clinically deficient variants of P450 17A1 have been identified (3, 4, 5). Some of the variants show normal 17α-hydroxylation activity but are deficient in the second, so-called “lyase,” reaction (Fig. 1), attenuating androgen production and subsequently estrogen levels. Conversely, prostate cancers are dependent on androgens (6), and the lyase reaction is an important drug target (7, 8, 9, 10, 11, 12, 13, 14, 15, 16, 17).

**Figure 1:**
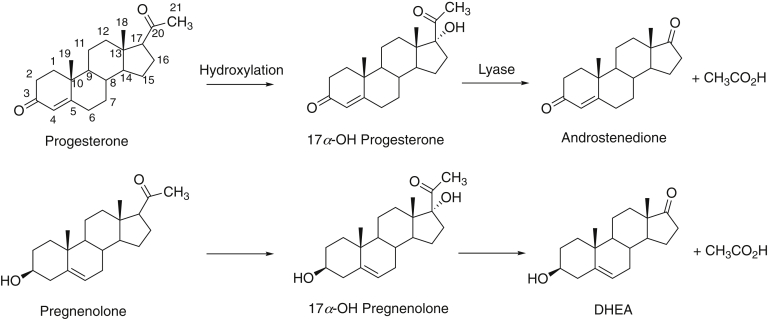
**Reactions catalyzed by P450 17A1.** The first reaction in each sequence is a 17α-hydroxylation, and the second reaction is a 17α, 20-cleavage reaction of the 21-carbon steroid (lyase reaction). DHEA, dehydroepiandrosterone.

Aside from its clinical relevance, P450 17A1 is of inherent biochemical interest in the context of the two reactions it catalyzes. The first reaction (Fig. 1) is a relatively straightforward P450 hydroxylation involving compound I (FeO^3+^) chemistry, but the mechanism of the second, the lyase step, is not without controversy (15, 18, 19). Although a number of mammalian P450 enzymes are stimulated by another hemeprotein, cytochrome *b*_5_ (CYB5A, *b*_5_), the P450 17A1 lyase reaction is the one stimulated the most (20, 21, 22, 23). This effect has been shown to be important biologically (*i.e.*, the interaction can be demonstrated using fluorescence resonance energy transfer in cells (24)), and deficient variants show low androgen levels *in vivo* (25, 26).

However, a phosphorylation event postulated to be at Ser-258 of P450 17A1 has also been proposed to facilitate the lyase reaction (27, 28, 29, 30), and that charge (negative) is opposite to that of the arginine residues (Arg-347 and Arg-358) proposed to be involved in binding *b*_5_ (3, 5, 31, 32, 33, 34, 35, 36). Zebrafish P450 17A1 lyase activity is only slightly enhanced by *b*_5_, and the related zebrafish P450 17A2 enzyme catalyzes only 17α-hydroxylation, not lyase activity, with or without *b*_5_ (37, 38). Mutation of residues to arginine in this region of zebrafish P450 17A2 did not lead to the acquisition of lyase activity (39).

One of the experimental deficiencies in this research field has been useful assays for *b*_5_ binding. We considered several approaches with fluorescence, in order to utilize sensitive and solution-based methods, and were able to use labeling of a previously described mutant with a dye (40, 41). Fluorescence attenuation assays allowed for analysis with submicromolar concentrations of proteins, with estimates of binding constants and rates of association and dissociation. In contrast to previous proposals about this system, we provide evidence that P450–*b*_5_ interactions are not tightly linked to substrate identity and occupancy and that a ternary P450 17A1–*b*_5_–NADPH–P450 reductase (POR) complex is preferred to a model in which *b*_5_ and POR shuttle at a single site.

## Results

### Expression and purification of P450 17A1 variant enzymes

**Figure 2:**
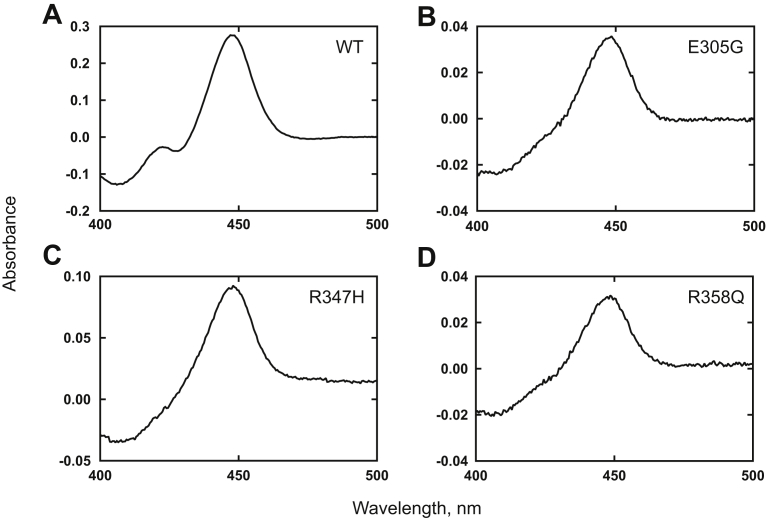
**Fe**^**2+**^**–CO *versus* Fe**^**2+**^**binding spectra of purified WT P450 17A1 and variant enzymes.***A*, WT; *B*, E305G; *C*, R347H; and *D*, R358Q. The P450 concentrations in these particular assays were 3.1, 0.41, 0.82, and 0.34 μM, respectively. These spectra are presented to demonstrate the lack of (inactive) cytochrome P420 and do not reflect the ratio of bound heme in the individual proteins.

Plasmids for WT P450 17A1 and five clinically observed variants were constructed in a pCW expression vector and expressed in *Escherichia coli* JM109 cells (Figs. 2 and S1). WT P450 17A1 showed an expression level of ∼450 nmol per liter culture, and the expression levels of the E305G, R347H, and R358Q variants were 400, 100, and 200 nmol per liter, respectively (Fig. S1). However, no P450 holoenzyme spectra were detected for the R347C and P428L variants (Fig. S1, *C* and *F*). Purified WT P450 17A1 and three variant proteins (E305G, R347H, and R358Q) were prepared by Ni^2+^–nitrilotriacetate (NTA) affinity column chromatography. The spectra of these purified variant proteins showed very little of peaks corresponding to inactive cytochrome P420 (Fig. 2, *B–D*). All subsequent assays were based on the amount of spectrally detectable P450 heme for WT and variant P450 17A1 enzymes.

### Steady-state kinetics of P450 17A1 variants

**Figure 3:**
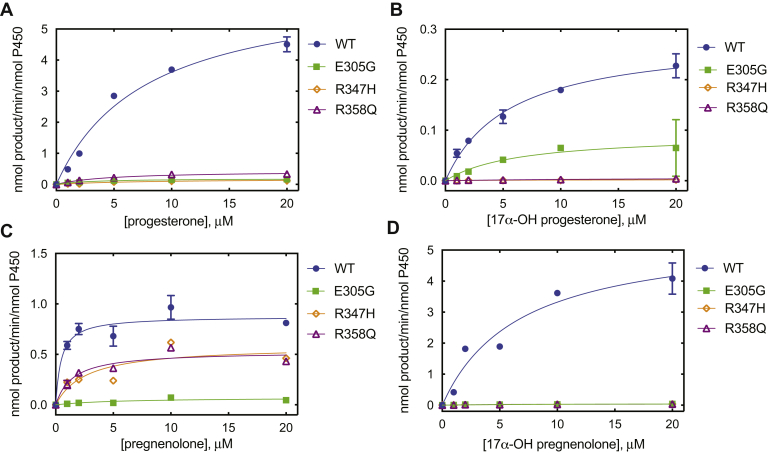
**Steady-state kinetics of enzyme reactions catalyzed by WT P450 17A1 and variants.***A*, WT (*blue*); *B*, E305G (*green*); *C*, R347H (*orange*); and *D*, R358Q (*purple*). Each point is a mean of duplicate assays, shown as a mean ± range. The kinetic parameters (*k*_cat_, *K*_*m*_, and *k*_cat_/*K*_*m*_) are indicated in Table 1.

Steady-state kinetic parameters for all (four) hydroxylation and lyase reactions (Fig. 1) of WT P450 17A1 and the three purified variants (E305G, R347H, and R358Q) were measured. For the 17α-hydroxylation of progesterone, all three variants formed the 17α-OH product, but their activities (specificity constants, *k*_cat_/*K*_*m*_) were attenuated to 3 to 11% of that of WT P450 17A1 (Fig. 3*A* and Table 1). The 17α-OH progesterone lyase activities of the R347H and R358Q variants were reduced more dramatically (to <1%) (Fig. 3*B* and Table 1). However, the E305G variant differed in that progesterone 17α-hydroxylation activity was decreased (to 8%) more than the lyase reaction activity (28%) (Fig. 3*A* and Table 1).

**Table 1:** Steady-state kinetic parameters for reactions of P450 17A1 variants

P450 17A1 variant	Progesterone 17α-hydroxylation				17α-OH progesterone conversion to androstenedione			
	*k*_cat_, min^−1^	*K*_*m*_, μM	*k*_cat_/*K*_*m*_	% WT (*k*_cat_/*K*_*m*_)	*k*_cat_, min^−1^	*K*_*m*_, μM	*k*_cat_/*K*_*m*_	% WT (*k*_cat_/*K*_*m*_)
WT	6.4 ± 0.5	7.8 ± 1.4	0.82 ± 0.16	100	0.29 ± 0.02	5.5 ± 0.8	0.052± 0.008	100
E305G	0.19 ± 0.01	3.1 ± 0.4	0.06 ± 0.01	8	0.092 ± 0.030	6.3 ± 5.1	0.015 ± 0.013	28
R347H	0.18 ± 0.02	8.1 ± 2.2	0.02 ± 0.01	4	0.002 ± 0.001	6.1 ± 1.2	0.0003 ± 0.002	0.6
R358Q	0.43 ± 0.02	4.6 ± 0.7	0.09 ± 0.02	11	0.006 ± 0.002	15 ± 9	0.0004 ± 0.0003	0.8

	**Pregnenolone 17α-hydroxylation**				**17α-OH pregnenolone conversion to DHEA**			

WT	0.88 ± 0.05	0.47 ± 0.20	1.9 ± 0.8	100	5.5 ± 0.7	6.7 ± 2.1	0.83 ± 0.28	100
E305G	0.07 ± 0.02	5.7 ± 4.6	0.01 ± 0.01	0.7	0.056 ± 0.010	9.4 ± 3.7	0.006 ± 0.003	0.7
R347H	0.53 ± 0.04	1.5 ± 0.5	0.35 ± 0.12	19	0.043 ± 0.006	4.8 ± 1.7	0.009 ± 0.003	1.1
R358Q	0.58 ± 0.09	2.6 ± 1.4	0.22 ± 0.13	12	0.056 ± 0.013	10 ± 5	0.005 ± 0.001	0.6

DHEA, dehydroepiandrosterone.

The R347H and R358Q variants showed similar levels of pregnenolone 17α-hydroxylation (slight reduction) but only very low levels of 17α-OH pregnenolone lyase activity (Fig. 3, *C* and *D* and Table 1). The E305G variant displayed highly reduced pregnenolone 17α-hydroxylation and 17α-OH pregnenolone lyase activities (Fig. 3, *C* and *D* and Table 1). One previous study reported a similar result (42), but another study did not (43). These results indicated that the 17,20-lyase activity in all three variants was impaired even when excess *b*_5_ was present.

### Substrate-binding affinities of P450 17A1 mutants

Substrate binding of purified P450 17A1 (WT and three variants) was analyzed in titrations with all four major substrates. WT P450 17A1 and all variant enzymes showed a typical “type І” substrate-binding spectral titration change (increase at 388 nm, decrease at 423 nm, corresponding to a shift in the iron spin state from low to high (44)) (Figs. S2 and S3). The calculated *K*_*d*_ value of WT P450 17A1 with progesterone was ∼0.065 μM, although even with quadratic analysis, there is considerable error in such a low value (Table 2). The *K*_*d*_ values of the three variants were somewhat increased, for example, from 0.17 to 0.26 μM (Table 2). The *K*_*d*_ values of variants for 17α-OH progesterone, pregnenolone, and 17α-OH pregnenolone were similar to or slightly higher than measured with WT P450 17A1 (Table 2). These results are interpreted to mean that changes in the substrate-binding affinities of the three variants are not primarily responsible for the decreased enzymatic activities, which were observed even at high substrate concentrations (Fig. 3).

**Table 2:** *K*_*d*_ values of P450 17A1·substrate complexes

P450 17A1 variant	Substrate			
	Progesterone, μM	17-OH progesterone, μM	Pregnenolone, μM	17-OH pregnenolone, μM
WT	0.065 ± 0.049	0.15 ± 0.01	0.14 ± 0.06	0.11 ± 0.05
E305G	0.17 ± 0.08	0.22 ± 0.17	0.26 ± 0.08	0.41 ± 0.18
R347H	0.23 ± 0.10	0.11 ± 0.12	0.12 ± 0.05	0.22 ± 0.10
R358Q	0.26 ± 0.12	0.17 ± 0.11	0.15 ± 0.06	0.26 ± 0.12

### *b*_*5*_ dependence of catalytic activities of P450 17A1

**Figure 4:**
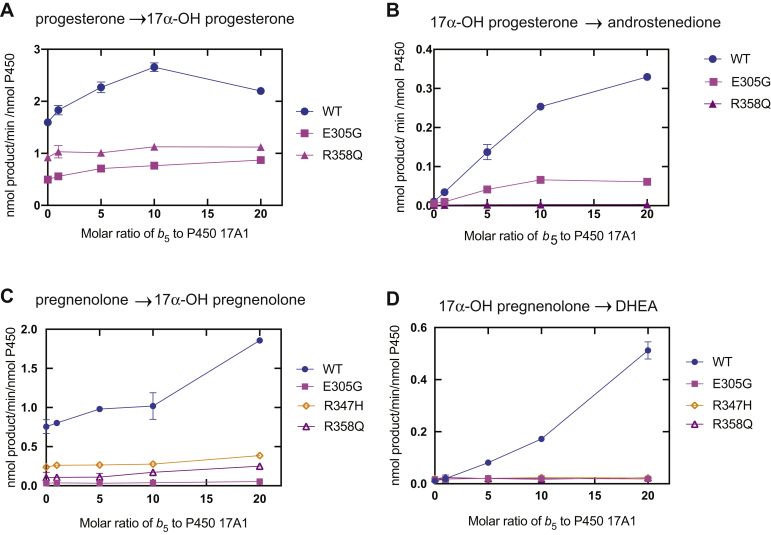
**Stimulation of reactions catalyzed by WT P450 17A1 and variants by *b***_**5**_**.***A*, WT (*blue*); *B*, E305G (*magenta*); *C*, R347H (*orange*); and *D*, R358Q (*purple*). Each point is a mean of duplicate assays, shown as a mean ± range.

The lyase catalytic activities of P450 17A1 are strongly stimulated by *b*_5_ (20, 21, 22, 23). *b*_5_ did not significantly stimulate the progesterone and pregnenolone 17α-hydroxylation reactions by WT P450 17A1 or the three variants (Fig. 4). WT P450 17A1 lyase activities were stimulated by *b*_5_ in a concentration-dependent manner (Fig. 4). However, the R347H and R358Q variants did not display *b*_5_ concentration-dependent stimulation in either of the lyase reactions. The E305G variant displayed weak *b*_5_ stimulation of the 17α-OH progesterone lyase reaction but not the 17α-OH pregnenolone lyase reaction (Fig. 4, *C* and *D*).

### Attenuation of Alexa 488-T70C-*b*_*5*_ fluorescence by WT P450 17A1 and variants

WT *b*_5_ has no cysteine residues, and the T70C substitution in *b*_5_ was chosen based on previous studies of fluorescent labeling with an acrylodan derivative and interactions of that modified protein with bacterial and other hemeproteins (40, 41). Preliminary labeling studies were done with NanoTemper reagents designed for microthermophoresis work (45). The proprietary “Red Reagent” was unsatisfactory for fluorescence titrations because of low sensitivity. The “Blue Reagent” provided much more sensitivity, and the resulting Blue-T70C-*b*_5_ protein stimulated the lyase reactions of (WT) P450 17A1 with both 17-OH progesterone and 17-OH pregnenolone as substrates, in a concentration-dependent manner (Fig. S4).

**Figure 5:**
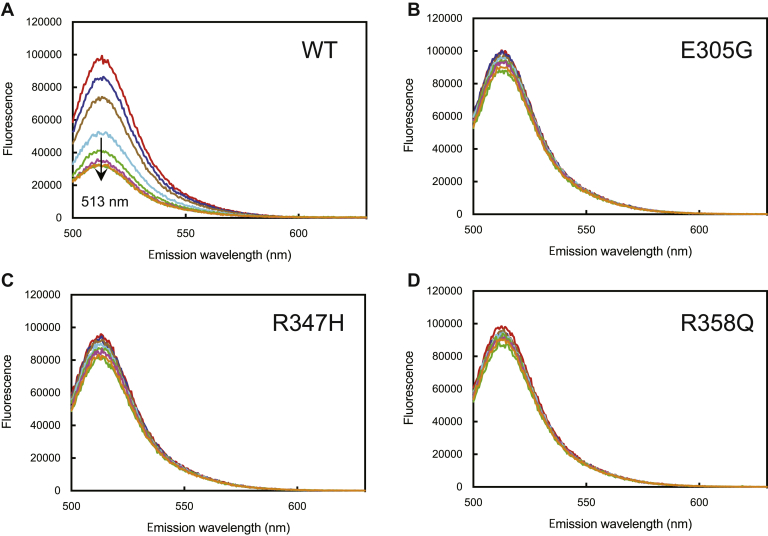
**Fluorescence titration of Alexa 488-T70C-*b***_**5**_**with WT P450 17A1 and variants.** Excitation of Alexa 488-T70C-*b*_5_ (50 nM) was at 480 nm. The emission spectra (*λ*_max_ of 513 nm) decreased with increasing concentrations of P450 17A1. *A*, WT; *B*, E305G; *C*, R347H; and *D*, R358Q. See Figure 6 for plots.

**Figure 6:**
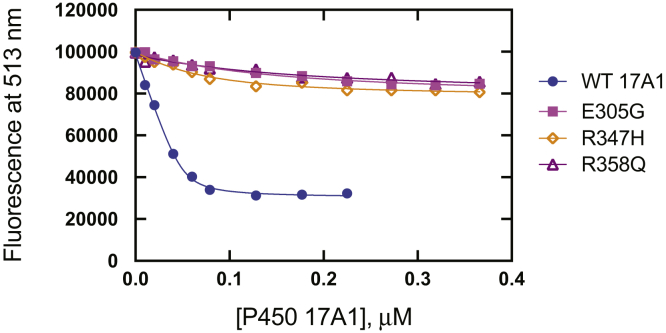
**Fitting of binding titrations of Alexa 488-T70C-*b***_**5**_**with P450 17A1 WT and variants.** The data points at the emission maximum (513 nm) in fluorescence titrations (Fig. 5) were fitted with a quadratic equation and nonlinear regression analysis in GraphPad Prism software: WT (*blue*, •); E305G (*magenta*, ▪); R347H (*orange*, ⋄); and R358Q (*purple*, ▵). The estimated *K*_*d*_ value for WT P450 17A1 was 2.5 ± 0.6 nM (Table 3).

The spectral properties of the Blue derivative suggested that the dye is Alexa 488 (or a closely related molecule), which is commercially available as a maleimide derivative for attachment to cysteine residues, and the protein modified with Alexa 488 (Alexa 488-T70C-*b*_5_) also stimulated the lyase reactions (results not shown). The fluorescence spectrum of Alexa 488-T70C-*b*_5_ showed an emission maximum at 513 nm (excitation wavelength of 480 nm) (Fig. 5), and the fluorescence emission displayed linearity with respect to the concentration of Alexa 488-T70C-*b*_5_ (Fig. S5). Titration of Alexa 488-T70C-*b*_5_ with WT P450 17A1 caused a loss of fluorescence intensity, indicating the perturbation of fluorescence by binding between Alexa 488-T70C-*b*_5_ and P450 17A1 (Fig. 5). Fitting of the loss of fluorescence with increased concentrations of P450 17A1 (using a quadratic equation) provided *K*_*d*_ values for the affinity of Alexa 488-T70C-*b*_5_ and P450 17A1 (Fig. 6 and Table 3). The apparent *K*_*d*_ value for WT P450 17A1 was ∼2.5 nM (Fig. 6 and Table 3), indicating very tight binding.

**Table 3:** *K*_*d*_ values of Alexa 488-T70C-*b*_5_:P450 17A1 complexes^a^

P450 17A1 variant	Substrate				
	No substrate, nM	Progesterone, nM	17-OH progesterone, nM	Pregnenolone, nM	17-OH pregnenolone, nM
WT	2.5 ± 0.6	5.8 ± 2.3	6.0 ± 1.0	12 ± 5	2.2 ± 1.0

a From Figure S6. See raw data for titrations with the variants (E305G, R347H, and R358Q).

With the three clinical variants, very little change in fluorescence spectra was observed in the binding titrations of Alexa 488-T70C-*b*_5_, indicating weak or no binding interaction between the *b*_5_ protein and the variant enzymes (Fig. 6). The effects of P450 17A1 substrates on the binding affinity of *b*_5_ and P450 17A1 were examined (Fig. S6 and Table 3). Very tight binding affinities (*K*_*d*_: 2.2–12 nM) were observed for WT P450 17A1/Alexa 488-T70C-*b*_5_ in the presence of each of the four substrates (present at 10 μM), not substantially altered from the binding of Alexa 488-T70C-*b*_5_ in the absence of substrate. The variants did not show binding in the presence or absence of substrates in the titrations (Fig. S6).

### Competition of POR with *b*_*5*_ for P450 17A1

**Figure 7:**
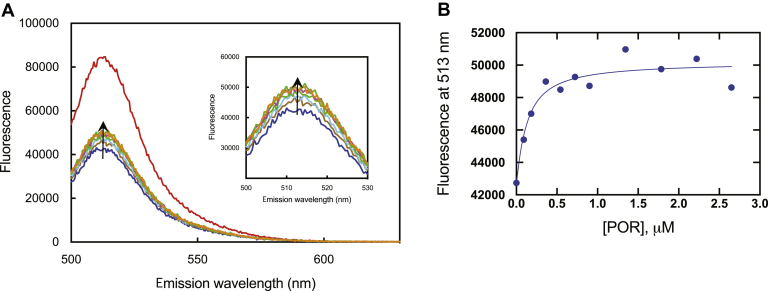
**Partial recovery of Alexa 488-T70C-*b***_**5**_**fluorescence of an Alexa 488-T70C-*b***_**5**_**:P450 17A1 complex by titration with POR**. *A*, the fluorescence of Alexa 488-T70C-*b*_5_ (50 nM) is shown with a *red trace*. Addition of P450 17A1 (50 nM) caused a drop in the fluorescence, to give the *blue trace*. The intensity of the fluorescence spectra increased upon adding increasing concentrations of POR (to 2.6 μM), in the direction shown with the *arrow*. *B*, recovery of fluorescence in the Alexa 488-T70C-*b*_5_:P450 17A1 complex as a function of POR concentration. The estimated *K*_*d*_ was 0.11 μM.

The loss of Alexa 488-T70C-*b*_5_ fluorescence induced by binding of P450 17A1 was partially reversed by titration with POR (Fig. 7). The P450 interaction of POR appeared to be competitive with Alexa 488-T70C-*b*_5_, but the original fluorescence values were never reached. The calculated *K*_*d*_ value of POR (for the WT P450 17A1:Alexa 488-T70C-*b*_5_ complex) was 0.11 μM, suggesting much lower affinity than *b*_5_ with P450 17A1 (Fig. 7).

### Binding rates for interaction of Alexa 488-T70C-*b*_*5*_ and P450 17A1

**Figure 8:**
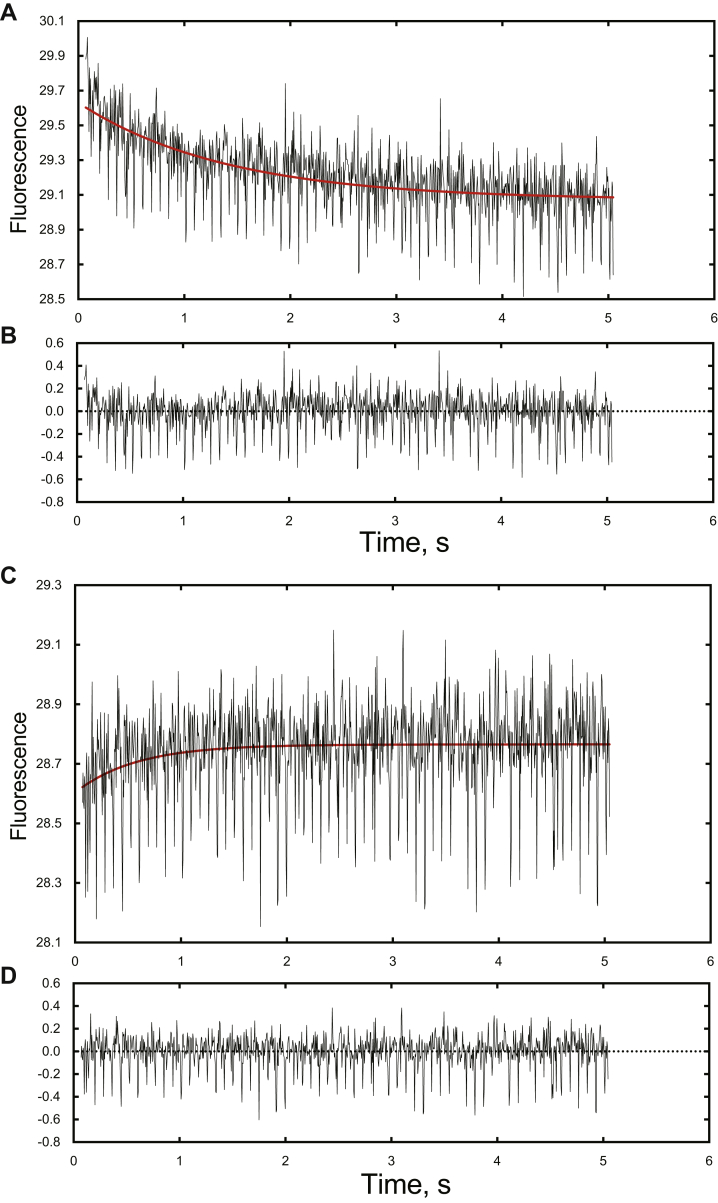
**Rates of binding and dissociation of Alexa 488-T70C-*b***_**5**_**and P450 17A1.***A*, stopped-flow analysis of binding of Alexa 488-T70C-*b*_5_ to P450 17A1 (1 μM concentrations of each protein in each syringe, excitation at 488 nm, emission >530 nm, and 23 °C). The decrease in fluorescence was fit to a single exponential using the OLIS GlobalWorks program (*red line*, 0.7 ± 0.1 s^−1^). *B*, residuals analysis for part *A*. *C*, stopped-flow analysis of binding of dissociation of Alexa 488-T70C-*b*_5_:P450 17A1. One syringe contained 100 nM Alexa 488-T70C-*b*_5_:P450 17A1, and the other contained 30 μM *b*_5_ (excitation at 488 nm and emission >530 nm). (When Alexa 488-T70C-*b*_5_ dissociated, it was replaced by *b*_5_.) The increase in fluorescence was fit to a single exponential using the OLIS GlobalWorks program (*red line*, 1.9 ± 0.2 s^−1^).

The rate of binding of Alexa 488-T70C-*b*_5_ to (WT) P450 17A1 was measured by observing attenuation of fluorescence upon mixing the two proteins in a stopped-flow fluorimeter. When concentrations of 0.50 μM Alexa 488-T70C-*b*_5_ and 0.50 μM P450 17A1 were mixed, the first-order *k*_obs_ value was 0.7 s^−1^ (Fig. 8, *A* and *B*).

The loss of Alexa 488-T70C-*b*_5_ fluorescence induced by binding of P450 17A1 was reversed by adding unlabeled *b*_5_ protein (data not presented), indicating that the unlabeled *b*_5_ replaces Alexa 488-T70C-*b*_5_ in interacting with P450 17A1. When the kinetics were measured, the reaction occurred at a rate of 1.9 s^−1^ (Fig. 8, *C* and *D*). This value is considered to be the first-order *k*_off_ rate.

In principle, for binding reactions in a freely reversible system, *k*_obs_ = *k*_on_ + *k*_off_ (46), but the apparent *k*_off_ rate here was higher than the *k*_obs_ (on) rate. Thus, the true *k*_on_ rate cannot be calculated from these measurements. However, the fluorescence traces clearly show that the binding and dissociation events are both occurring with rates of ∼1 s^−1^.

### Interaction of peptides with Alexa 488-T70C-*b*_*5*_ and P450 17A1

**Figure 9:**
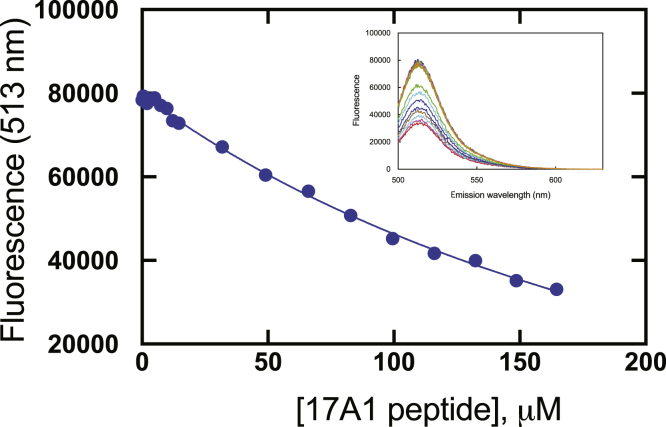
**Attenuation of the fluorescence of Alexa 488-T70C-*b***_**5**_**by a peptide corresponding to the putative binding region of P450 17A1.** Increasing concentrations of the peptide were added. The inset shows the fluorescence at 513 nm as a function of concentration. The concentration of Alexa 488-T70C-*b*_5_ was 50 nM.

Peptides were obtained corresponding to the putative binding sites of P450 17A1 and *b*_5_ (see Experimental procedures section and Fig. S7). The peptide corresponding to the putative *b*_5_ binding region of P450 17A1 (residues 347–358, “P450 peptide”) attenuated the fluorescence of Alexa 488-T70C-*b*_5_, although high concentrations were required (Fig. 9). The “*b*_5_ peptide,” however, did not result in a gain of fluorescence when added to an Alexa 488-T70C-*b*_5_:P450 17A1 complex, even at a concentration of 160 μM (data not presented).

**Figure 10:**
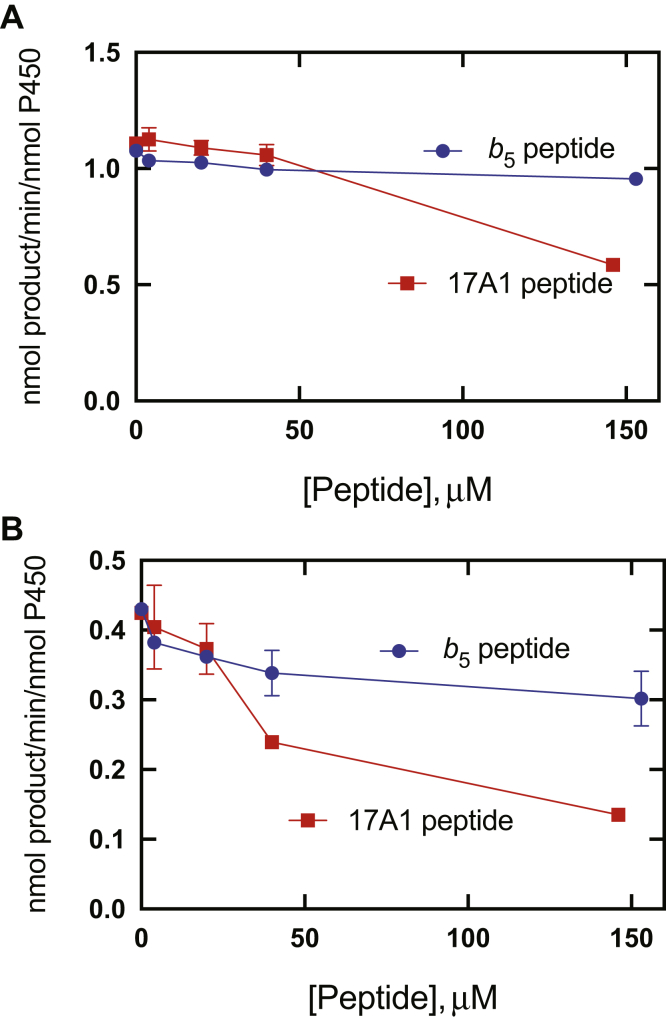
**Inhibition of P450 17A1 reactions by peptides corresponding to the putative binding regions of P450 17A1 and *b***_**5**_**.***A*, progesterone 17α-hydroxylation; *B*, 17α-OH pregnenolone lyase activity. The “*b*_5_ peptide” corresponds to residues 43 to 52 of *b*_5_, and the “17A1 peptide” corresponds to residues 347 to 358 of P450 17A1 (see Experimental procedures section and Fig. S7).

When added to reconstituted P450 17A1 steroid oxidation systems (Fig. 10), the P450 17A1 peptide was more inhibitory to the lyase reaction (17α-OH pregnenolone) than the *b*_5_ peptide, which may appear to be surprising in that *b*_5_ was present in the reaction at a concentration 10-fold higher than the P450. This peptide might be expected to bind to POR and block its interaction with P450 17A1, although that should have also inhibited the 17α-hydroxylation reaction. Inhibition was only observed at the very highest concentration.

### Demonstration of a ternary P450 17A1:POR:*b*_5_ ternary complex using size-exclusion chromatography

**Figure 11:**
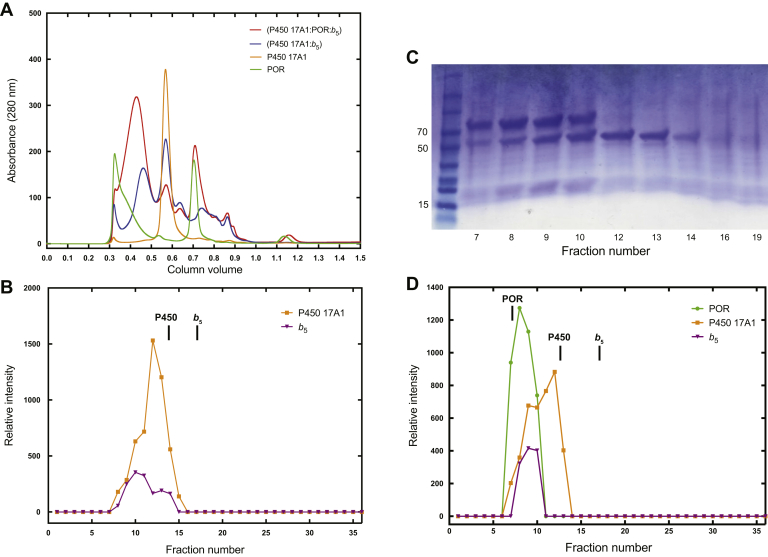
**Gel filtration analysis of complexes of P450 17A1, *b***_**5**_**, and POR.** All analyses were done using a Superose 12 10/300 FPLC column. *A*, absorbance at 280 nm profiles of individual proteins (POR [*green*] and P450 17A1 [*orange*]) and a binary (*blue*) and a ternary mixture (*red*) are shown. Individual fractions were collected and analyzed by SDS-polyacrylamide gel electrophoresis, and densitometry was done of the Coomassie Blue–stained bands corresponding to the individual proteins. The POR preparation contained unknown 280 nm-absorbing material eluting near the position of free *b*_5_ but not showing any protein after electrophoresis and staining. *B*, densitometry traces of P450 17A1 (*orange*) and *b*_5_ (*purple*) eluted in a binary equimolar mixture of the two proteins. The migration positions of the individual proteins (P450 [17A1] and *b*_5_) are indicated. *C*, Coomassie Blue staining of the proteins in a ternary complex, as eluted from the column in Part *A*. The numbers on the left indicate *M*_r_ values of markers relevant to the three proteins of interest, which have approximate *M*_r_s of 79 kDa (POR), 57 kDa (P450 17A1), and 17 kDa (*b*_5_). *D*, densitometry traces of P450 17A1 (*orange*), POR (*green*), and *b*_5_ (*purple*) eluted in a ternary equimolar mixture of the three proteins. The migration positions of the individual proteins (POR, P450 [17A1], and *b*_5_) are indicated.

The fluorescence titration results (Fig. 7) suggested that P450 17A1, POR, and *b*_5_ form a ternary complex. Accordingly, we tested this hypothesis further using a different approach, that is, gel filtration (Fig. 11*A*). In a previous work with P450 17A1 (47), we reported that about one-half of the protein migrated as a monomer, but in this case, all the P450 17A1 eluted as a single monomeric peak on a Superose 12 column, at an elution volume between those of ovalbumin (45 kDa) and bovine serum albumin (67 kDa, monomer) (Fig. S8). *b*_5_ eluted later, as might be expected, and POR eluted as a multimer near the void volume of the column (Fig. 11*A*). (The identity of the second peak in the POR sample is unknown and presumed to be a small molecule, in that no proteins were visualized upon SDS-gel electrophoresis and Coomassie Blue staining; Fig. 11*B*).

A complex of P450 17A1 and *b*_5_ yielded peaks in the monomeric P450 17A1 and *b*_5_ regions plus a larger complex eluting earlier, as verified with gel electrophoresis (Fig. 11, *A* and *B*). A mixture of POR, P450 17A1, and *b*_5_ had most of the 280 nm-absorbing material (protein) in a large peak eluting later than free POR but earlier than the P450 17A1–*b*_5_ complex, as validated by gel electrophoresis (Fig. 11, *A*, *C*, and *D*). The presence of all three proteins in the ternary complex peak fractions (Fig. 11, *A* and *D*) is documented in Figure 11*C*.

## Discussion

The development of a sensitive fluorescence-based assay for *b*_5_ binding, based on a literature precedent with an unrelated system (40, 41), enabled assays that could address several issues in the field of P450 17A1. A major finding was the very tight binding of *b*_5_ to P450 17A1, with an estimated *K*_*d*_ of ∼2.5 nM (Fig. 6 and Table 2). Comparisons with P450 17A1 variants known to be deficient in lyase activity (3, 31, 43) and documented here (Table 1) showed the importance of the P450 17A1 residues Arg-347 and Arg-358, previously proposed to be involved in the interaction (3, 5, 31, 32, 33, 34, 35, 36). The binding phenomena we studied are attributed to the ionic charges in P450 17A1 and *b*_5_, in that all binding assays were done in the absence of phospholipid vesicles.

**Figure 12:**
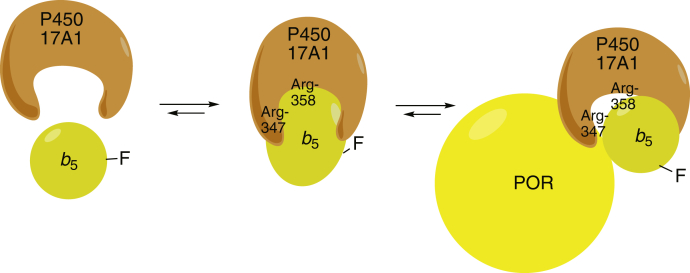
**Proposed scheme for interaction of P450 17A1, *b***_**5**_**, and POR.** The fluorescence of Alexa 488-T70C-*b*_5_ (F depicts the fluorophore Alexa 488) is decreased upon interaction with P450 17A1. POR binds to form a ternary complex and causes a shift of the fluorophore (Alexa 488) to a position in which the fluorescence is partially restored (Fig. 7).

Stopped-flow measurements with P450 17A1 showed that both the binding of *b*_5_ and its dissociation are rapid processes. The rates are on the order of 1 s^−1^, although the *k*_obs_ value for binding (Fig. 8*A*) is probably not accurate in that it should be greater than the (first-order) dissociation rate (Fig. 8*C*). The *k*_obs_ value for binding is probably a reflection of a faster “on” rate followed by a conformational change to a complex in which the fluorescence is decreased (46). The *k*_obs_ values for binding and dissociation are fast enough not to be rate limiting in the overall P450 17A1 oxidation reactions (Table 1). However, the results can be used to argue against a model in which POR and *b*_5_ are “switching” at the same position on P450 17A1 during catalysis, unless this would be facilitated in the phospholipid vesicles. That is, the (substrate-bound) P450 Fe^2+^O_2_ complex needs to receive an electron, thus converting it to the “compound 0” form, Fe^3+^–O_2_^–^. If POR donated the electron, then it would have to leave and allow the *b*_5_ to bind (*k* ∼ 1 s^−1^) (48) and induce a conformational change to permit the lyase reaction. It is unlikely that compound 0 would have enough stability for this to occur (*i.e.*, a *k*_obs_ of 1 s^−1^ is equivalent to a *t*_1/2_ of 0.7 s). The bulk of the evidence is that *b*_5_ does not donate the second electron in the reaction (24, 33, 47, 49, 50), even if it can to some extent in the results of Duggal *et al*. (51, 52). Furthermore, lyase activity was stimulated in mammalian cells in which apo-*b*_5_ was expressed, demonstrating the biological relevance of these findings (53). In the event that *b*_5_ were donating the second electron, the Fe^2+^O_2_ complex would have to be stable enough for the binding to occur after POR left the P450. We conclude that our proposal of a ternary P450:*b*_5_:POR complex (Figs. 7, 11, and 12) describes the mechanism more accurately than does a shuttle system.

The fluorescence attenuation titrations (Figs. 5 and 6) were critical in development of the conclusions about the tight binding of *b*_5_ to (WT) P450 17A1. The attenuation of fluorescence in the presence of P450 17A1 cannot be attributed to an inner filter effect, because of the low absorbance, the reversal of the decrease by *b*_5_ (Fig. 8*C*) or a second protein (POR), and the lack of attenuation by the three P450 17A1 variants, even at the highest P450 concentrations. The *K*_*d*_ values (Table 3) should be considered estimates and have some uncertainty associated with them, for several reasons. With WT P450 17A1, the value is very low, and even with a low concentration of Alexa 488-T70C-*b*_5_ (50 nM) and a quadratic equation, there is probably error in the *K*_*d*_ value (2.5 nM, *i.e.*, 20-fold less than the enzyme concentration) (Table 2). With regard to the variants, they clearly did not attenuate the Alexa 488-T70C-*b*_5_ fluorescence much (Fig. 5), but any *K*_*d*_ calculations have the caveat that we do not know exactly what the titration endpoint should be, that is, a complex formed with Alexa 488-T70C-*b*_5_ and one of the variants may have the *b*_5_ (and the Alexa 488 dye) in a position in which it does not show as much loss of fluorescence.

A molecular description of the fluorescence of the Alexa 488 derivative of *b*_5_ is beyond the scope of this investigation. Glu-48 and Glu-49 are on the edge of *b*_5_ (Protein Data Bank: 2I96), and the heme is between these residues and Thr-70. Stayton *et al*. (40, 41) attached the fluorescent dye acrylodan, which has a very different structure than Alexa 488, to Cys-65, engineered to replace Thr-65 in rat *b*_5_ (corresponding to human Thr-70). The fluorescence of this derivative increased upon binding to metmyoglobin, cytochrome *c*, or (bacterial) P450_cam_, with estimates of *K*_*d*_ of ∼1 μM for each. Blue shifts in the emission spectrum were also observed (40), which is known to be very sensitive to changes in dielectric constants. We have not prepared the acrylodan derivative of T70C *b*_5_ for use with P450 17A1. In other work (not shown), the fluorescence of Alexa 488-T70C-*b*_5_ was attenuated by several other human P450 enzymes (*e.g.*, 2C9, 2E1, 3A4), but the *K*_*d*_ values were not as low as with P450 17A1. Apparently, the heme of P450 (17A1) is not absolutely required for fluorescence attenuation, in that the P450 17A1 peptide was able to reduce the fluorescence (Fig. 9), albeit with weaker affinity. Preliminary screening studies have also identified several small molecules (at 10 μM concentration) that can attenuate the fluorescence of Alexa 488-T70C-*b*_5_ (results not presented). Whether the heme of *b*_5_ is critical for the attenuation of fluorescence by small molecules of proteins is unknown, in that we have not studied the apo-*b*_5_ version of the conjugate. Another issue is any conformational changes that occur upon P450 17A1–*b*_5_ binding. The stepwise addition of P450 17A1 to ^15^N-labeled *b*_5_ induced some NMR shift perturbations, which may be indicative of conformational changes (in *b*_5_), although the Thr-70 signal did not appear to be changed (36). Exactly how a conformational change, if it occurs, would effect the fluorescence is unknown in the absence of more information.

Our fluorescence results are in agreement with earlier reports on the roles of Arg-347 and Arg-358 in *b*_5_ binding and the basis of loss of lyase activity (3, 32, 33, 34, 35, 36, 42). We also agree with Ershov *et al*. (54) that binding of *b*_5_ to P450 17A1 is relatively tight. However, our results lead to several different conclusions about the interaction of P450 17A1 and *b*_5_, primarily because of the sensitivity of the assays (Figs. 5 and 6), which involve the components in solution.

The *K*_*d*_ values for binding of substrates to P450 17A1 (Table 2) are all lower than previously reported from this laboratory (23). The main reason is that the previous results were based on assays involving endpoints of stopped-flow measurements of binding rates, and the values reported here were obtained in steady-state assays with lower P450 concentrations (Fig. S3 and Table 2). Although the *K*_*d*_ values for substrate binding were somewhat higher with some of the variants (Table 2), attenuated substrate binding is not the major reason for low activity, and activities were low even at high concentrations of substrate (Fig. 3). The results of the work with E305G are not consistent with some of the conclusions of Sherbet *et al*. (43), who used an indirect approach to characterizing substrate binding. As discussed later, the basis of loss of activity of the E305G variant is more complex than the other variants.

In contrast to the reports of Estrada *et al*. (36, 55), who used NMR measurements at very high enzyme concentrations, we did not find a major effect of any substrate on *b*_5_ binding (Figs. S2 and S3 and Table 2). Our results are consonant with our published work indicating that the presence of *b*_5_ did not alter rates of substrate binding or dissociation (23, 47).

The surface plasmon resonance (SPR) results of Ershov *et al*. (54) also show relatively low *K*_*d*_ values for *b*_5_ binding, but these values are an order of magnitude higher that our own (Table 3). The SPR method involves immobilization of a protein on a chip, and a major deficiency of the approach is “mass transfer,” a term used to describe the diffusion of the ligand from the solution through the matrix to reach the receptor (P450 17A1 in this case) (46). The reported on-rate constants (54) are extremely low (*i.e.*, 360–5000 M^−1^ s^−1^) for the binding of a substrate to an enzyme, as is usually the case in SPR work, and are not in line with our own on- and off-rates (Fig. 8), even if our on-rates may not be completely accurate.

A key finding of our study is that POR only partially disrupted the Alexa 488-T70C-*b*_5_:P450 17A1 complex (Fig. 7). If all the Alexa 488-T70C-*b*_5_ had become displaced by POR and completely free, the fluorescence would have reverted to the original value. The hyperbolic nature of the binding (Fig. 7*B*) could be used to calculate a *K*_*d*_, with multiple equilibria if there were complete displacement, but if a ternary complex is formed, then the level of residual fluorescence may not be estimated. If there is a ternary complex, then the observed *K*_*d*_ for binding the POR (110 nM) is valid (∼25 times the *K*_*d*_ for *b*_5_). Mixing POR with Alexa 488-T70C-*b*_5_ did not decrease its fluorescence, although interaction must exist between those two proteins in that POR reduces *b*_5_ (56).

The gel filtration results provide more evidence for the existence of a ternary P450 17A1–POR–*b*_5_ complex (Fig. 11). The elution profile of a 17A1–*b*_5_ complex was shifted relative to either monomer and the complex of a 1:1:1 M mixture of all three proteins yielded a new peak that contained all three proteins (Fig. 11, *C* and *D*). These results, along with the fluorescence results presented in Figure 7, are consistent with a ternary complex (Fig. 12). The complex is dynamic, as shown by the kinetic assays of P450 17A1–*b*_5_ binding (Fig. 8) but stable enough to persist during migration in a gel filtration column (Fig. 11). This model of a ternary complex (Fig. 12) differs from a shuttle mechanism developed on the basis of NMR measurements (36, 57). Our results and our model are also probably not consistent with the model of Holien *et al*. (58), in which *b*_5_ and POR bind to the far ends of a P450 17A1 dimer (in a ternary complex) but are not in contact with each other. However, it is conceivable that, in such a model, the binding of POR on one end of a P450 dimer could transduce a fluorescence increase of the *b*_5_ on the other end. A shuttle mechanism with POR and *b*_5_ alternately binding transiently to P450 has also been proposed for (rabbit) P450 2B4 (59, 60) and human P450 3A4 (61), but at this time, we cannot speculate on the general application of this model (Fig. 12) to other P450s that use *b*_5_.

The P450 17A1 E305G variant is complex in that, in our assays, it lost most of the catalytic activities, with the exception of only partial loss of 17α-OH progesterone conversion to androstenedione (Fig. 3*B*). These results contrast with a previous report using a different system (heterologous expression in yeast and use of yeast microsomes), in which the lower lyase activity was attributed to attenuated substrate binding (43). We did not observe altered substrate binding (Table 2 and Figs. S2 and S3). In addition, the E305G variant did not quench the fluorescence of Alexa 488-T70C-*b*_5_, which we interpret as lack of binding of *b*_5_, even though Glu-305 is not at the face of P450 17A1 thought to bind directly to *b*_5_. Our current hypothesis is that the E305G substitution leads to a conformational change in P450 17A1 that disrupts *b*_5_ binding (Figs. 4, *B* and *D* and 5*B*).

Although we have provided new information about the nature of the interaction of P450 17A1 and *b*_5_, a number of issues and questions still remain. A sensitive interaction assay is described and applied, but there may be other dyes that could provide even greater sensitivity. As mentioned previously, the E305G variant is more complex than previously thought, and the charge distant from the putative *b*_5_ binding site has an effect on binding. Also, this variant retained some lyase activity with 17α-OH progesterone but not 17α-OH pregnenolone (Figs. 3 and 4), even though *b*_5_ binding was poor (Figs. 5*B* and 6). Finally, if our conclusion about a ternary complex (of P450 17A1, *b*_5_, and POR) is correct, we do not know the spatial relationship of the proteins.

As mentioned earlier, a phosphorylation event postulated to be at Ser-258 of P450 17A1 has been proposed to facilitate the lyase reaction (27, 28, 29, 30), and that charge (negative) is opposite to that of the arginine residues. What has not been clear from that work is whether the phosphorylation is sufficient in itself to stimulate the lyase reaction or whether the phosphorylation enhances the functional binding of *b*_5_ to produce the enhancement. Resolving the issue would require the purification of a specifically phosphorylated P450 17A1 or the analysis of activity in a system (cells?) with a *b*_*5*_^−/−^ background.

In teleost fish, there are two P450 17A enzymes, 17A1 and 17A2 (62). Fish P450 17A1 enzymes resemble human P450 17A1 and catalyze both 17α-hydroxylation and lyase activities, but P450 17A2 only catalyzes the former reaction. Zebrafish P450 17A1 showed only a twofold stimulation of the lyase activity by human or zebrafish *b*_5_, and P450 17A2 shows no activity with or without *b*_5_ (37). The exact basis of the lyase deficiency in fish P450 17A2 remains unknown, and the X-ray crystal structures of the two proteins are very similar (37). In contrast to fish, *Xenopous laevis* (frog) appears to have only P450 17A1, not 17A2 (UniProt search). Although recombinant *X. laevis* P450 17A1 androgen biosynthesis has been suggested to be independent of *b*_5_ (38), a closer examination of that report and calculation of *k*_cat_/*K*_m_ values indicates that lyase activity was stimulated 12-fold by *X. laevis b*_5_ in the case of 17α-OH pregnenolone and 1.5-fold in the case of 17α-OH progesterone. The extent of *b*_5_ stimulation is difficult to reach conclusions about in such systems where P450 17A1 and POR are present in microsomal membranes and soluble *b*_5_ is added, in that the *b*_5_ may not have the same access to the P450 as seen in a more homogeneous or even a vesicular system, although stimulations are qualitatively seen in systems with *b*_5_ added to yeast microsomes (3, 38).

The results with the peptides (Fig. 10) showed weak inhibition of the lyase activity by the P450 17A1 peptide but inhibition of progesterone 17α-hydroxylation only at the very highest concentration of that peptide. In principle, it might be possible to develop drugs that block binding of *b*_5_ to P450 17A1 to discover selective inhibitors of the lyase reactions, an unmet need in treating prostate cancer (14, 15, 17).

In conclusion, we have developed a sensitive fluorescence assay that can be utilized to study *b*_5_ interactions with P450 17A1. We confirm previous conclusions that Arg-347 and Arg-358 of P450 17A1 are important in *b*_5_ binding. We also implicated loss of *b*_5_ binding in the low activity of the P450 17A1 variant E305G. In contrast to previous work, we did not observe effects of P450 17A1 on its interactions with *b*_5_, and we provide evidence for a functional P450 17A1–POR–*b*_5_ ternary complex instead of a shuttle mechanism.

## Experimental procedures

### Chemicals and enzymes

Progesterone, pregnenolone, 17-OH progesterone, 17-OH pregnenolone, androstenedione, dehydroepiandrosterone, protease inhibitor cocktail, cholesterol oxidase, and 1,2-dilauroyl-*sn*-glycero-3-phosphocholine were purchased from Sigma–Aldrich. Ni^2+^–NTA–agarose was purchased from Qiagen. 5-Aminolevulinic acid was purchased from Frontier Scientific. IPTG and CHAPS were purchased from Anatrace. Other chemicals were of the highest grade commercially available. Peptides were purchased from New England Peptides, with purity and identity analysis provided (Fig. S8):



with the numbering of the residues shown from the primary sequence.

### Enzyme expression and purification

*E. coli* JM109 cells were purchased from Invitrogen. Recombinant rat POR and human *b*_5_ were expressed in *E. coli* and purified as described previously (56, 63, 64, 65).

Expression and purification of the P450 17A1 enzymes and variants was carried out as previously described but with some modifications (66, 67). Briefly, *E. coli* JM109 cells transformed using pCW (Ori^+^) vectors were inoculated into terrific broth medium containing 100 μg ml^−1^ ampicillin, 0.5 mM 5-aminolevulinic acid, and 1.0 mM IPTG. The expression cultures (500 ml) were grown at 37 °C for 3 h and then at 30 °C under conditions of shaking at 200 rpm for 28 h in 2.8-l Fernbach flasks. Soluble fractions containing P450 17A1 enzymes were prepared after ultracentrifugation (10^5^*g*, 60 min). The soluble fraction was then loaded onto a Ni^2+^–NTA column (Qiagen), and (after washing), the purified protein was eluted with 100 mM potassium phosphate buffer (pH 7.4) containing 0.5 M NaCl, 0.5% (w/v) CHAPS, 20% (v/v) glycerol, and 250 mM imidazole. The eluted fraction containing highly purified P450 17A1 was dialyzed at 4 °C against 100 mM potassium phosphate buffer (pH 7.4) containing 20% (v/v) glycerol and 0.1 mM EDTA to remove CHAPS and NaCl.

Fe^2+^–CO *versus* Fe^2+^ binding spectra were recorded using the method of Omura and Sato (68), using an extinction coefficient of 91,000 M^−1^ cm^−1^ (Δε_450–490_). Protein concentrations were estimated from *A*_280_ measurements using a Nanodrop spectrophotometer (Thermo Scientific) and the amino acid compositions of human P450 17A1 and *b*_5_. The estimated specific content of WT P450 17A1 was 7.3 nmol P450 (mg protein)^−1^, which is somewhat low but within the historical range of 5.6 to 16.8 nmol P450 (mg protein)^−1^ for purified P450s expressed in *E. coli* and purified in this laboratory (69, 70, 71, 72, 73). The estimated specific contents of the purified E305G, R437H, and R358Q variants were 5.5, 2.3, and 4.2 nmol P450 (mg protein)^−1^, respectively. We have observed heme contents as low as 10% of the WT values for expressed variants of other human P450s, for example, P450 21A2 (74). All catalytic and titration assays comparing the WT and variant P450 17A1 enzymes were done on the basis of spectrally detected P450 heme, though (as before (74)), in order to avoid differences because of heme content.

### Construction of Alexa 488-T70C-*b5*

The expression plasmid for T70C-*b*_5_ was constructed using an Agilent quikChange site-directed mutagenesis kit according to the manufacturer's instructions. The constructed mutant plasmid was verified with DNA nucleotide sequencing analysis and then transformed into *E. coli* DH5α. The purification was performed with the same procedure for WT *b*_5_ previously described (56). The calculated specific content of *b*_5_ heme in T70C-*b*_5_ was 59.9 nmol (mg protein)^−1^, within experimental error of the theoretical amount for a protein of 16.9 kDa.

The purified protein was labeled using a NanoTemper Monolith Protein Labeling Kit BLUE NHS second Generation (NanoTemper Technologies), which reacts with sulfhydryl groups in a protein sample to label the protein with a fluorescent dye (BLUE) (proprietary). The extent of labeling was determined by a modification of the manufacturer instructions.

Labeling of the T70C-*b*_5_ mutant was then performed using a maleimide conjugation reaction with Alexa Fluor 488 dye (Thermo Scientific). Briefly, the *b*_5_ T70C mutant (in 100 mM phosphate buffer, pH 7.4) was mixed with Alexa Fluor 488 maleimide (dissolved in dimethylsulfoxide) at a 1:10 M ratio, and the conjugation reaction was allowed to proceed at room temperature for 20 h in the dark. The labeling mixture was then passed through a Zeba spin column (Thermo Scientific) to eliminate unreacted dye. The concentration of (labeled) Alexa 488-T70C-*b*_5_ was calculated by measuring the absorbance, using *ε*_493_ = 72,000 M^−1^ cm^−1^. The extent of modification was 74%.

### Substrate-binding analysis

Binding titration analysis was carried out using purified WT P450 17A1 and variant enzymes to determine the binding affinity parameters of the substrate–enzyme complexes. The purified enzymes were diluted in 100 mM potassium phosphate buffer (pH 7.4) and divided in two 1.0-ml glass cuvettes. Spectra (350–500 nm) were recorded using an OLIS-DW2 spectrophotometer (On-Line Instrument Systems) with subsequent additions of the substrate. Differences in the absorbance between the wavelength maximum and minimum were plotted *versus* the substrate concentration (44). *K*_*d*_ values were calculated using nonlinear regression analysis in GraphPad Prism software (GraphPad) and the quadratic equation:

$$Y=B\;+\;\frac{A}{2E}[\left(K_{d}\;+\;E\;+\;X\right)-\sqrt{{\left(K_{d}\;+\;E\;+\;X\right)}^{2}-\;4EX}\;]\text{,}$$

set in Prism as: Y = B + (A/2) ∗ (1/E) ∗ ((Kd + E + X)-sqrt((Kd + E + X)^ˆ^2 - (4 ∗ E ∗ X))).

### P450 catalytic activity analysis

P450 catalytic activity assays to measure steroid hydroxylation and lyase reactions were carried out as described previously (23, 37, 64). P450 17A1 enzymes were reconstituted and preincubated in 500 μl of 100 mM potassium phosphate buffer (pH 7.4) containing POR, 1,2-dilauroyl-*sn*-glycero-3-phosphocholine, and the substrate (progesterone, 17-OH progesterone, pregnenolone, or 17-OH pregnenolone). Typical enzyme concentrations used were P450 17A1 (0.1–0.2 μM), POR (0.4–0.8 μM), and *b*_5_ (2–4 μM) for hydroxylation/lyase reactions (*e.g.*, Figs. 3 and 4 and Table 1).

The reactions were initiated by adding an NADPH-regenerating system (final concentrations of 15 mM glucose 6-phosphate, 1.5 mM NADP^+^, and 1 IU ml^−1^ glucose 6-phosphate dehydrogenase) (75) and terminated by quenching and extracting with 2.0 ml CH_2_Cl_2_. Aliquots of the organic phases were dried and then dissolved in 100 μl of a CH_3_CN/H_2_O (1:1, v/v) mixture. The extracts from the pregnenolone and 17-OH pregnenolone reactions were dissolved in 50 μl of CH_3_OH, mixed with 200 μl of cholesterol oxidase (0.5 units/reaction) in 100 mM phosphate buffer (pH 7.4), and incubated at 30 °C with shaking at 150 rpm for 12 h. The extraction procedure was then repeated, and the (Δ^4^) reaction products were analyzed in a Waters Acquity UPLC system using a BEH C18 octadecylsilane column (2.1 mm × 100 mm, 1.7 μm). The reaction products were resolved using a mobile phase composed of solvents A (70% CH_3_OH, 30% H_2_O, v/v) and B (CH_3_CN), at a flow rate of 0.2 ml min^−1^. The mobile phase linear gradient proceeded as follows: 0 to 1 min, 5% B; 4 min, 30% B; 4.5 min, 40% B; 4.55 to 6.75 min, 95% BA; and 7 to 10 min, 5% B. The reaction products were identified by coelution with commercial standards and quantified by the *A*_243_ peak areas. Steady-state kinetic parameters were estimated using nonlinear regression of hyperbolic fits in GraphPad Prism software, with equations used for *k*_cat_ and the specificity constant *k*_cat_/*K*_*m*_ (76): Y = (ksp ∗ X)/(1 + (ksp ∗ X/kcat)) and then deriving *K*_*m*_ by division.

### P450 titrations with fluorescent *b*_5_

Steady-state fluorescence spectra were collected on an OLIS DM-45 spectrofluorimeter. For optimal fluorescence measurements, 1.24 mm slits (corresponding to a 5.0 nm bandwidth) were employed. The purified Alexa 488-T70C-*b*_5_ protein was diluted to 50 nM in 100 mM potassium phosphate buffer (pH 7.4) in a 1.0-ml cuvette. Binding titrations were carried out using increasing concentrations of WT P450 17A1 and variant enzymes. Emission spectra (500–630 nm) were recorded after subsequent additions of P450 enzymes (excitation wavelength of 480 nm). The data points at the emission maximum (513 nm) were used for analysis with a quadratic equation and nonlinear regression analysis in GraphPad Prism software, set as Y = B + (A/2) ∗ (1/E) ∗ ((*K*_*d*_ + E + X)-sqrt((*K*_*d*_ + E + X)ˆ2-(4 ∗ E ∗ X))).

### Association and dissociation kinetics of Alexa 488-T70C-*b*_*5*_ and P450 17A1

Association kinetics of *b*_5_ and P450 17A1 were measured using a stopped-flow spectrofluorimeter (RSM-1000; On-Line Instrument Systems) equipped with a 4 × 4 mm cell (23 °C). The two drive syringes were filled with 1.0 μM Alexa 488-T70C-*b*_5_ protein and 1.0 μM P450 17A1 (in 100 mM potassium phosphate buffer, pH 7.4), respectively. After mixing, the instrument recorded the emission >530 nm (Orion long-pass filter) with an excitation wavelength of 488 nm, over a period of 5 s. To estimate the dissociation rate (*k*_off_), unlabeled *b*_5_ (30 μM) in one syringe was mixed with a preformed complex of Alexa 488-T70C-*b*_5_ (1.0 μM) and P450 17A1 (1.0 μM) in the other syringe. Rates were estimated using GlobalWorks software analysis of the experimental data, fitting to a single exponential (data from at least three replicates were averaged.)

### Size-exclusion chromatography

Chromatography was done using a Superose 12 10/300 GL column (11 μm, 10 × 300 mm; GE Healthcare) with an NGC Quest 100 Plus Chromatography system (BioRad). The buffer was 50 mM potassium phosphate (pH 7.4) containing 0.15 M NaCl, and the flow rate was 1.0 ml min^−1^. The column was equilibrated for each run with one column volume (23.6 ml), and the injection volume was 3% of the column volume (10 nmol of each protein was injected, *i.e.*, 100 μl of 100 μM solutions). Elution was with 1.5 column volumes of buffer. Absorbance was monitored at 280 nm. Fractions were collected (1.0 ml) and analyzed by SDS-polyacrylamide gel electrophoresis (4–15% gradient gel), with staining with Coomassie Blue and densitometry using GelAnalyzer 19.1 software (www.gelanalyzer.com; Istvan Lazar and Istvan Lazar Jr). *M*_r_ standards included ovalbumin and bovine serum albumin (Fig. S9).

## Data availability

All data are contained within the article and the supporting information.

## Supporting information

This article contains supporting information.

## Conflict of interest

The authors declare that they have no conflict of interest with the contents of this article.

## References

[1] Rendic S., Guengerich F.P. (2015). Survey of human oxidoreductases and cytochrome P450 enzymes involved in the metabolism of xenobiotic and natural chemicals. Chem. Res. Toxicol..

[2] Auchus R.J., Miller W.L., Ortiz de Montellano P.R. (2015). P450 enzymes in steroid processing. Cytochrome P450: Structure, Mechanism, and Biochemistry.

[3] Geller D.H., Auchus R.J., Miller W.L. (1999). P450c17 mutations R347H and R358Q selectively disrupt 17,20-lyase activity by disrupting interactions with P450 oxidoreductase and cytochrome *b*_5_. Mol. Endocrinol..

[4] DeVore N.M., Scott E.E. (2012). Structures of cytochrome P450 17A1 with prostate cancer drugs abiraterone and TOK-001. Nature.

[5] Auchus R.J. (2017). Steroid 17-hydroxylase and 17,20-lyase deficiencies, genetic and pharmacologic. J. Steroid Biochem. Mol. Biol..

[6] Huggins C., Stevens R.E. (1940). The effect of castration on benign hypertrophy of the prostate in man. J. Urol..

[7] de Bono J.S., Logothetis C.J., Molina A., Fizazi K., North S., Chu L., Chi K.N., Jones R.J., Goodman O.B., Saad F., Staffurth J.N., Mainwaring P., Harland S., Flaig T.W., Hutson T.E. (2011). Abiraterone and increased survival in metastatic prostate cancer. N. Engl. J. Med..

[8] Loriot Y., Bianchini D., Ileana E., Sandhu S., Patrikidou A., Pezaro C., Albiges L., Attard G., Fizazi K., De Bono J.S., Massard C. (2013). Antitumour activity of abiraterone acetate against metastatic castration-resistant prostate cancer progressing after docetaxel and enzalutamide (MDV3100). Ann. Oncol.

[9] Yin L., Hu Q. (2014). CYP17 inhibitors–abiraterone, C17,20-lyase inhibitors and multi-targeting agents. Nat. Rev. Urol..

[10] Scott L.J. (2017). Abiraterone acetate: A review in metastatic castration-resistant prostrate cancer. Drugs.

[11] Stein M.N., Goodin S., Dipaola R.S. (2012). Abiraterone in prostate cancer: A new angle to an old problem. Clin. Cancer Res..

[12] Pinto-Bazurco Mendieta M.A., Negri M., Jagusch C., Muller-Vieira U., Lauterbach T., Hartmann R.W. (2008). Synthesis, biological evaluation, and molecular modeling of abiraterone analogues: Novel CYP17 inhibitors for the treatment of prostate cancer. J. Med. Chem..

[13] Yang L.P. (2011). Abiraterone acetate: In metastatic castration-resistant prostate cancer. Drugs.

[14] Wróbel T.M., Rogova O., Andersen K.L., Yadav R., Brixius-Anderko S., Scott E.E., Olsen L., Jørgensen F.S., Björkling F. (2020). Discovery of novel non-steroidal cytochrome P450 17A1 inhibitors as potential prostate cancer agents. Int. J. Mol. Sci..

[15] Burris-Hiday S.D., Scott E.E. (2021). Steroidogenic cytochrome P450 17A1 structure and function. Mol. Cell. Endocrinol..

[16] Saad F., Fizazi K., Jinga V., Efstathiou E., Fong P.C., Hart L.L., Jones R., McDermott R., Wirth M., Suzuki K., MacLean D.B., Wang L., Akaza H., Nelson J., Scher H.I. (2015). Orteronel plus prednisone in patients with chemotherapy-naive metastatic castration-resistant prostate cancer (ELM-PC 4): A double-blind, multicentre, phase 3, randomised, placebo-controlled trial. Lancet Oncol..

[17] Krug S.J., Hu Q., Hartmann R.W. (2013). Hits identified in library screening demonstrate selective CYP17A1 lyase inhibition. J. Steroid Biochem. Mol. Biol..

[18] Bhatt M.R., Khatri Y., Rodgers R.J., Martin L.L. (2017). Role of cytochrome *b*_5_ in the modulation of the enzymatic activities of cytochrome P450 17α-hydroxylase/17,20-lyase (P450 17A1). J. Steroid Biochem. Mol. Biol..

[19] Guengerich F.P., Yoshimoto F.K. (2018). Formation and cleavage of C-C bonds by enzymatic oxidation-reduction reactions. Chem. Rev..

[20] Katagiri M., Kagawa N., Waterman M.R. (1995). The role of cytochrome *b*_5_ in the biosynthesis of androgens by human P450c17. Arch. Biochem. Biophys..

[21] Onoda M., Hall P.F. (1982). Cytochrome *b*_5_ stimulates purified testicular microsomal cytochrome P-450 (C21 side-chain cleavage). Biochem. Biophys. Res. Commun..

[22] Katagiri M., Suhara K., Shiroo M., Fujimura Y. (1982). Role of cytochrome *b*_5_ in the cytochrome P-450-mediated C21-steroid 17,20-lyase reaction. Biochem. Biophys. Res. Commun..

[23] Gonzalez E., Guengerich F.P. (2017). Kinetic processivity of the two-step oxidations of progesterone and pregnenolone to androgens by human cytochrome P450 17A1. J. Biol. Chem..

[24] Simonov A.N., Holien J.K., Yeung J.C., Nguyen A.D., Corbin C.J., Zheng J., Kuznetsov V.L., Auchus R.J., Conley A.J., Bond A.M., Parker M.W., Rodgers R.J., Martin L.L. (2015). Mechanistic scrutiny identifies a kinetic role for cytochrome *b*_5_ regulation of human cytochrome P450c17 (CYP17A1, P450 17A1). PLoS One.

[25] Kok R.C., Timmerman M.A., Wolffenbuttel K.P., Drop S.L., de Jong F.H. (2010). Isolated 17,20-lyase deficiency due to the cytochrome *b*_5_ mutation W27X. J. Clin. Endocrinol. Metab..

[26] Idkowiak J., Randell T., Dhir V., Patel P., Shackleton C.H., Taylor N.F., Krone N., Arlt W. (2012). A missense mutation in the human cytochrome *b*_5_ gene causes 46,XY disorder of sex development due to true isolated 17,20 lyase deficiency. J. Clin. Endocrinol. Metab..

[27] Tee M.K., Dong Q., Miller W.L. (2008). Pathways leading to phosphorylation of P450c17 and to the posttranslational regulation of androgen biosynthesis. Endocrinology.

[28] Wang Y.H., Tee M.K., Miller W.L. (2010). Human cytochrome P450c17: Single step purification and phosphorylation of serine 258 by protein kinase A. Endocrinology.

[29] Tee M.K., Miller W.L. (2013). Phosphorylation of human cytochrome P450c17 by p38α selectively increases 17,20 lyase activity and androgen biosynthesis. J. Biol. Chem..

[30] Pandey A.V., Miller W.L. (2005). Regulation of 17,20 lyase activity by cytochrome *b*_5_ and by serine phosphorylation of P450c17. J. Biol. Chem..

[31] Geller D.H., Auchus R.J., Mendonça B.B., Miller W.L. (1997). The genetic and functional basis of isolated 17,20-lyase deficiency. Nat. Genet..

[32] Peng H.M., Liu J., Forsberg S.E., Tran H.T., Anderson S.M., Auchus R.J. (2014). Catalytically relevant electrostatic interactions of cytochrome P450c17 (CYP17A1) and cytochrome *b*_5_. J. Biol. Chem..

[33] Lee-Robichaud P., Akhtar M.E., Akhtar M. (1998). Control of androgen biosynthesis in the human through the interaction of Arg^347^ and Arg^358^ of CYP17 with cytochrome *b*_5_. Biochem. J..

[34] Lee-Robichaud P., Akhtar M.E., Akhtar M. (1999). Lysine mutagenesis identifies cationic charges of human CYP17 that interact with cytochrome *b*_5_ to promote male sex-hormone biosynthesis. Biochem. J..

[35] Lee-Robichaud P., Akhtar M.E., Wright J.N., Sheikh Q.I., Akhtar M. (2004). The cationic charges on Arg347, Arg358 and Arg449 of human cytochrome P450c17 (CYP17) are essential for the enzyme's cytochrome *b*_5_-dependent acyl-carbon cleavage activities. J. Steroid Biochem. Mol. Biol..

[36] Estrada D.F., Laurence J.S., Scott E.E. (2013). Substrate-modulated cytochrome P450 17A1 and cytochrome *b*_5_ interactions revealed by NMR. J. Biol. Chem..

[37] Pallan P.S., Nagy L.D., Lei L., Gonzalez E., Kramlinger V.M., Azumaya C.M., Wawrzak Z., Waterman M.R., Guengerich F.P., Egli M. (2015). Structural and kinetic basis of steroid 17α,20-lyase activity in teleost fish cytochrome P450 17A1 and its absence in cytochrome P450 17A2. J. Biol. Chem..

[38] Yang W.H., Hammes S.R. (2005). *Xenopus laevis* CYP17 regulates androgen biosynthesis independent of the cofactor cytochrome *b*_5_. J. Biol. Chem..

[39] Gonzalez E., Johnson K.M., Pallan P.S., Phan T.T.N., Zhang W., Lei L., Wawrzak Z., Yoshimoto F.K., Egli M., Guengerich F.P. (2018). Inherent steroid 17α,20-lyase activity in defunct cytochrome P450 17A enzymes. J. Biol. Chem..

[40] Stayton P.S., Poulos T.L., Sligar S.G. (1989). Putidaredoxin competitively inhibits cytochrome *b*_5_-cytochrome P-450_cam_ association: A proposed molecular model for a cytochrome P-450_cam_ electron-transfer complex. Biochemistry.

[41] Stayton P.S., Fisher M.T., Sligar S.G. (1988). Determination of cytochrome *b*_5_ association reactions: Characterization of metmyoglobin and cytochrome P-450_cam_ binding to genetically engineered cytochrome *b*_5_. J. Biol. Chem..

[42] Peng H.M., Im S.C., Pearl N.M., Turcu A.F., Rege J., Waskell L., Auchus R.J. (2016). Cytochrome *b*_5_ activates the 17,20-lyase activity of human cytochrome P450 17A1 by increasing the coupling of NADPH consumption to androgen production. Biochemistry.

[43] Sherbet D.P., Tiosano D., Kwist K.M., Hochberg Z., Auchus R.J. (2003). CYP17 mutation E305G causes isolated 17,20-lyase deficiency by selectively altering substrate binding. J. Biol. Chem..

[44] Schenkman J.B., Remmer H., Estabrook R.W. (1967). Spectral studies of drug interaction with hepatic microsomal cytochrome P-450. Mol. Pharmacol..

[45] Child S.A., Reddish M.J., Glass S.M., Goldfarb M.H., Barckhausen I.R., Guengerich F.P. (2020). Functional interactions of adrenodoxin with several human mitochondrial cytochrome P450 enzymes. Arch. Biochem. Biophys..

[46] Johnson K.A. (2019). Kinetic Analysis for the New Enzymology.

[47] Guengerich F.P., Wilkey C.J., Glass S.M., Reddish M.J. (2019). Conformational selection dominates binding of steroids to human cytochrome P450 17A1. J. Biol. Chem..

[48] Mak P.J., Gregory M.C., Denisov I.G., Sligar S.G., Kincaid J.R. (2015). Unveiling the crucial intermediates in androgen production. Proc. Natl. Acad. Sci. U. S. A..

[49] Auchus R.J., Lee T.C., Miller W.L. (1998). Cytochrome *b*_5_ augments the 17,20-lyase activity of human P450c17 without direct electron transfer. J. Biol. Chem..

[50] Brock B.J., Waterman M.R. (1999). Biochemical differences between rat and human cytochrome P450c17 support the different steroidogenic needs of these two species. Biochemistry.

[51] Duggal R., Liu Y., Gregory M.C., Denisov I.G., Kincaid J.R., Sligar S.G. (2016). Evidence that cytochrome *b*_5_ acts as a redox donor in CYP17A1 mediated androgen synthesis. Biochem. Biophys. Res. Commun..

[52] Duggal R., Denisov I.G., Sligar S.G. (2018). Cytochrome *b*_5_ enhances androgen synthesis by rapidly reducing the CYP17A1 oxy-complex in the lyase step. FEBS Lett..

[53] Storbeck K.H., Swart A.C., Lombard N., Adriaanse C.V., Swart P. (2012). Cytochrome *b*_5_ forms homomeric complexes in living cells. J. Steroid Biochem. Mol. Biol..

[54] Ershov P.V., Yablokov E.O., Florinskaya A.V., Mezentsev Y.V., Kaluzhskiy L.A., Tumilovich A.M., Gilep A.A., Usanov S.A., Ivanov A.S. (2019). SPR-based study of affinity of cytochrome P450s/redox partners interactions modulated by steroidal substrates. J. Steroid Biochem. Mol. Biol..

[55] Estrada D.F., Skinner A.L., Laurence J.S., Scott E.E. (2014). Human cytochrome P450 17A1 conformational selection: Modulation by ligand and cytochrome *b*_5_. J. Biol. Chem..

[56] Guengerich F.P. (2005). Reduction of cytochrome *b*_5_ by NADPH-cytochrome P450 reductase. Arch. Biochem. Biophys..

[57] Estrada D.F., Laurence J.S., Scott E.E. (2016). Cytochrome P450 17A1 interactions with the FMN domain of its reductase as characterized by NMR. J. Biol. Chem..

[58] Holien J.K., Parker M.W., Conley A.J., Corbin C.J., Rodgers R.J., Martin L.L. (2017). A homodimer model can resolve the conundrum as to how cytochrome P450 oxidoreductase and cytochrome *b*_5_ compete for the same binding site on cytochrome P450c17. Curr. Protein Pept. Sci..

[59] Zhang H., Im S.C., Waskell L. (2007). Cytochrome *b*_5_ increases the rate of product formation by cytochrome P450 2B4 and competes with cytochrome P450 reductase for a binding site on cytochrome P450 2B4. J. Biol. Chem..

[60] Im S.C., Waskell L. (2011). The interaction of microsomal cytochrome P450 2B4 with its redox partners, cytochrome P450 reductase and cytochrome *b*_5_. Arch. Biochem. Biophys..

[61] Zhao C., Gao Q., Roberts A.G., Shaffer S.A., Doneanu C.E., Xue S., Goodlett D.R., Nelson S.D., Atkins W.M. (2012). Cross-linking mass spectrometry and mutagenesis confirm the functional importance of surface interactions between CYP3A4 and holo/apo cytochrome *b*_5_. Biochemistry.

[62] Zhou L.Y., Wang D.S., Kobayashi T., Yano A., Paul-Prasanth B., Suzuki A., Sakai F., Nagahama Y. (2007). A novel type of P450c17 lacking the lyase activity is responsible for C21-steroid biosynthesis in the fish ovary and head kidney. Endocrinology.

[63] Hanna I.H., Teiber J.F., Kokones K.L., Hollenberg P.F. (1998). Role of the alanine at position 363 of cytochrome P450 2B2 in influencing the NADPH- and hydroperoxide-supported activities. Arch. Biochem. Biophys..

[64] Park H.G., Lim Y.R., Han S., Jeong D., Kim D. (2017). Enhanced purification of recombinant rat NADPH-P450 reductase by using a hexahistidine-tag. J. Microbiol. Biotechnol..

[65] Kim D., Cryle M.J., De Voss J.J., Ortiz de Montellano P.R. (2007). Functional expression and characterization of cytochrome P450 52A21 from *Candida albicans*. Arch. Biochem. Biophys..

[66] Yoshimoto F.K., Gonzalez E., Auchus R.J., Guengerich F.P. (2016). Mechanism of 17α,20-lyase and new hydroxylation reactions of human cytochrome P450 17A1: ^18^O labeling and oxygen surrogate evidence for a role of a perferryl oxygen. J. Biol. Chem..

[67] Lim Y.R., Han S., Kim J.H., Park H.G., Lee G.Y., Le T.K., Yun C.-H., Kim D. (2017). Characterization of a biflaviolin synthase CYP158A3 from *Streptomyces avermitilis* and its role in the biosynthesis of secondary metabolites. Biomol. Ther. (Seoul).

[68] Omura T., Sato R. (1964). The carbon monoxide-binding pigment of liver microsomes. I. Evidence for its hemoprotein nature. J. Biol. Chem..

[69] Sandhu P., Baba T., Guengerich F.P. (1993). Expression of modified cytochrome P450 2C10 (2C9) in *Escherichia coli*, purification, and reconstitution of catalytic activity. Arch. Biochem. Biophys..

[70] Guo Z., Gillam E.M., Ohmori S., Tukey R.H., Guengerich F.P. (1994). Expression of modified human cytochrome P450 1A1 in *Escherichia coli*: Effects of 5′ substitution, stabilization, purification, spectral characterization, and catalytic properties. Arch. Biochem. Biophys..

[71] Wu Z.L., Sohl C.D., Shimada T., Guengerich F.P. (2006). Recombinant enzymes overexpressed in bacteria show broad catalytic specificity of human cytochrome P450 2W1 and limited activity of human cytochrome P450 2S1. Mol. Pharmacol..

[72] Sohl C.D., Guengerich F.P. (2010). Kinetic analysis of the three-step steroid aromatase reaction of human cytochrome P450 19A1. J. Biol. Chem..

[73] Albertolle M.E., Song H.D., Wilkey C.J., Segrest J.P., Guengerich F.P. (2019). Glutamine-451 confers sensitivity to oxidative inhibition and heme-thiolate sulfenylation of cytochrome P450 4B1. Chem. Res. Toxicol..

[74] Wang C., Pallan P.S., Zhang W., Lei L., Yoshimoto F.K., Waterman M.R., Egli M., Guengerich F.P. (2017). Functional analysis of human cytochrome P450 21A2 variants involved in congenital adrenal hyperplasia. J. Biol. Chem..

[75] Guengerich F.P., Hayes A.W., Kruger C.L. (2014). Analysis and characterization of enzymes and nucleic acids relevant to toxicology. Hayes' Principles and Methods of Toxicology.

[76] Johnson K.A. (2019). New standards for collecting and fitting steady state kinetic data. Beilstein J. Org. Chem..

