# Supplementary material for: Tight binding of cytochrome b5 to cytochrome P450 17A1 is a critical feature of stimulation of C21 steroid lyase activity and androgen synthesis
Source: J Biol Chem. 2021 Mar 20;296:100571. doi: 10.1016/j.jbc.2021.100571 (PMC8080067; doi:10.1016/j.jbc.2021.100571)
Supplement: Figures S1 to S8 [file mmc1.docx]

**Supporting Information**

**Tight binding of cytochrome *b*_5_ to cytochrome P450 17A1 is a critical feature of stimulation of C21 steroid lyase activity and androgen synthesis**

Donghak Kim^1,2^, Vitchan Kim^2^, Kevin D. McCarty^1^, and F. Peter Guengerich^1^

From the ^1^Department of Biochemistry, Vanderbilt University School of Medicine, Nashville, Tennessee 37232-0146 U. S. A. and ^2^Department of Biological Sciences, Konkuk University, Seoul 05029, Republic of Korea

**Table of Contents**

Figure S1. Expression of WT P450 17A1 and variants.

Figure S2. Titrations of WT P450 17A1 and variants with substrates–spectra.

Figure S3. Titrations of WT P450 17A1 and variants with substrates–binding plots.

Figure S4. Stimulation of lyase activities by *b*_5_, T70C- *b*_5_, and NanoTemper Blue-T70C-*b*_5_.

Figure S5. Linearity of fluorescence response of Alexa 488-T70C-*b*_5_.

Figure S6. Titrations of Alexa 488-T70C-*b*_5_ with WT P450 17A1 and three variants in the presence of substrates.

Figure S7. Identity and purity of P450 and *b*_5_ binding region peptides.

Figure S8. Elution of P450 17A1 and ovalbumin and BSA standards from a Superose 12 gel filtration column.

**Figure S1. Expression of WT P450 17A1 and variants.** Ferrous-CO vs. ferrous binding spectra of wild-type P450 17A1 and variants in *Escherichia coli* whole cells were measured (at varying dilutions), and the contents of the P450 holoenzyme were calculated: *A*, wild-type, 450 nmol P450/liter culture; *B*, E305G, 400 nmol P450/liter; *D*, R347H, 100 nmol P450/liter; and *E*, R358Q, 200 nmol P450/liter. No holoenzyme content was observed in Parts *C*, R347C, and *F*, P428L.

**Figure S2. Titrations of WT P450 17A1 and variants with substrates–spectra.** Increasing concentrations of substrates were added to the sample and reference cuvettes in each case. The P450 concentration was 1.0 µM in each case. Δ*A*_388-_*A*_423_ *vs.* the concentration of each substrate was plotted and *K*_d_ values were calculated (Fig. S3, Table 2). *A*, progesterone; *B*, 17α-OH progesterone; *C*, pregnenolone; *D*, 17α-OH pregnenolone.

**Figure S3. Titrations of WT P450 17A1 and variants with substrates–binding plots.** Plots (hyperbolic-quadratic) of the data from Fig. S2. *A*, progesterone; *B*, 17α-OH progesterone; *C*, pregnenolone; *D*, 17α-OH pregnenolone.

**Figure S4. Stimulation of lyase activities by *b*_5_, T70C- *b*_5_, and NanoTemper Blue-T70C-*b*_5_.** Catalytic assays for the lyase reactions were performed and analyzed as described in the main text. Increasing concentrations of WT- *b*_5_, T70C-*b*_5_, and NanoTemper Blue-T70C-*b*_5_ were added to reaction mixtures including 10 μM of 17α-OH progesterone or 17α-OH pregnenolone as substrate.

**Figure S5. Linearity of fluorescence response of Alexa 488-T70C-*b*_5_.** The fluorescence emission intensity at 513 nm (excitation 480 nm) was measured with increasing concentrations of Alexa 488-T70C-*b*_5_. Fitting was analyzed using Graph-Pad Prism software (linear regression).

**Figure S6. Titrations of Alexa 488-T70C-*b*_5_ with WT P450 17A1 and three variants in the presence of substrates.** Binding titrations were carried out using purified wild-type P450 17A1 and variant enzymes including 10 μM of each substrate. Emission spectra (500-630 nm; excitation wavelength 480 nm) were recorded after subsequent additions of WT P450 17A1 and variant enzymes to a solution of 50 nM Alexa 488-T70C-*b*_5_. *A*, progesterone; *B*, 17α-OH progesterone; *C*, pregnenolone; *D*, 17α-OH pregnenolone.

**A P450 17A1 peptide**

347 358

R N R L L L L E A T I R


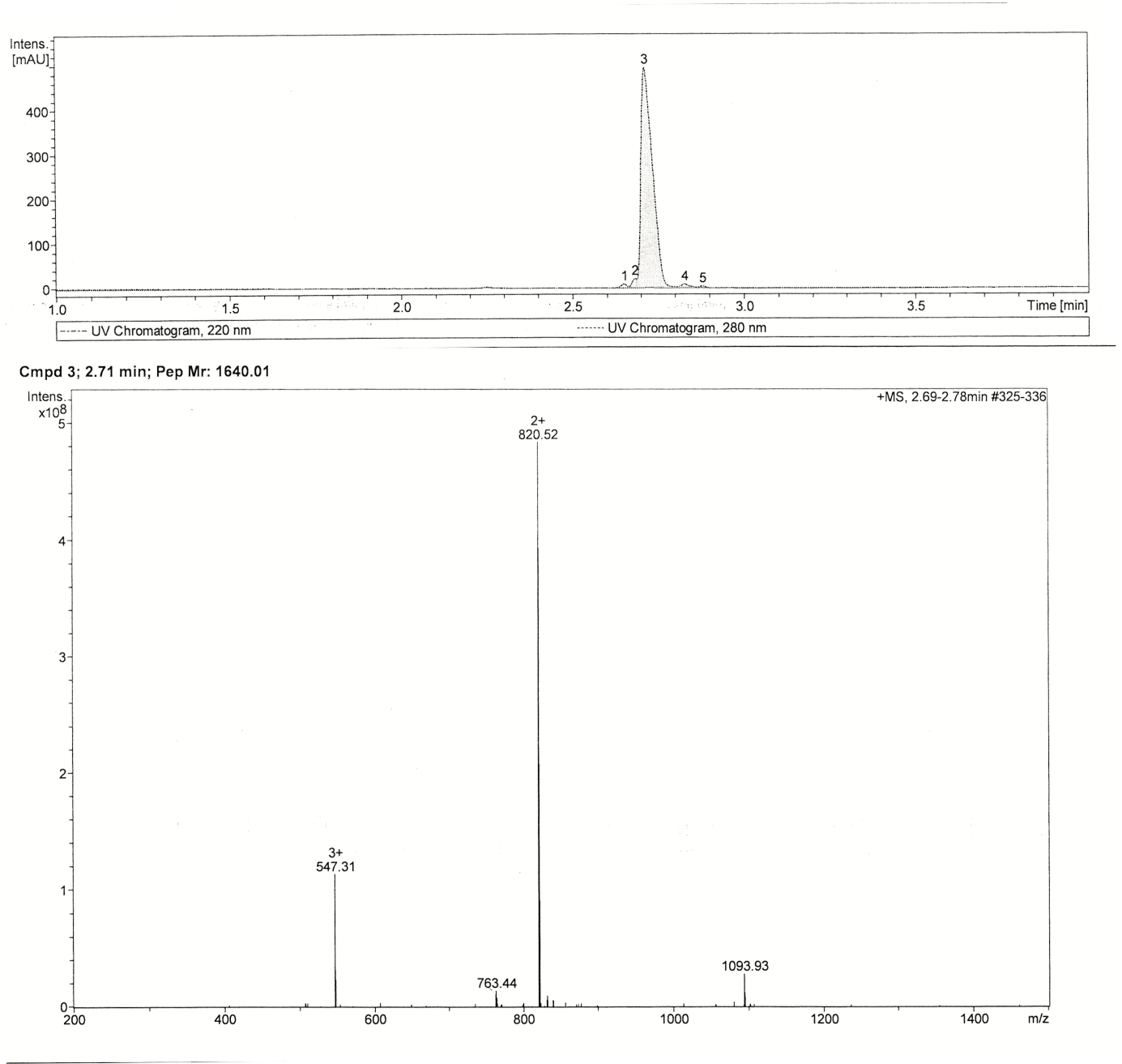


**B *b*_5_ peptide**

48 49 52

E H P G G E E V L R E

**
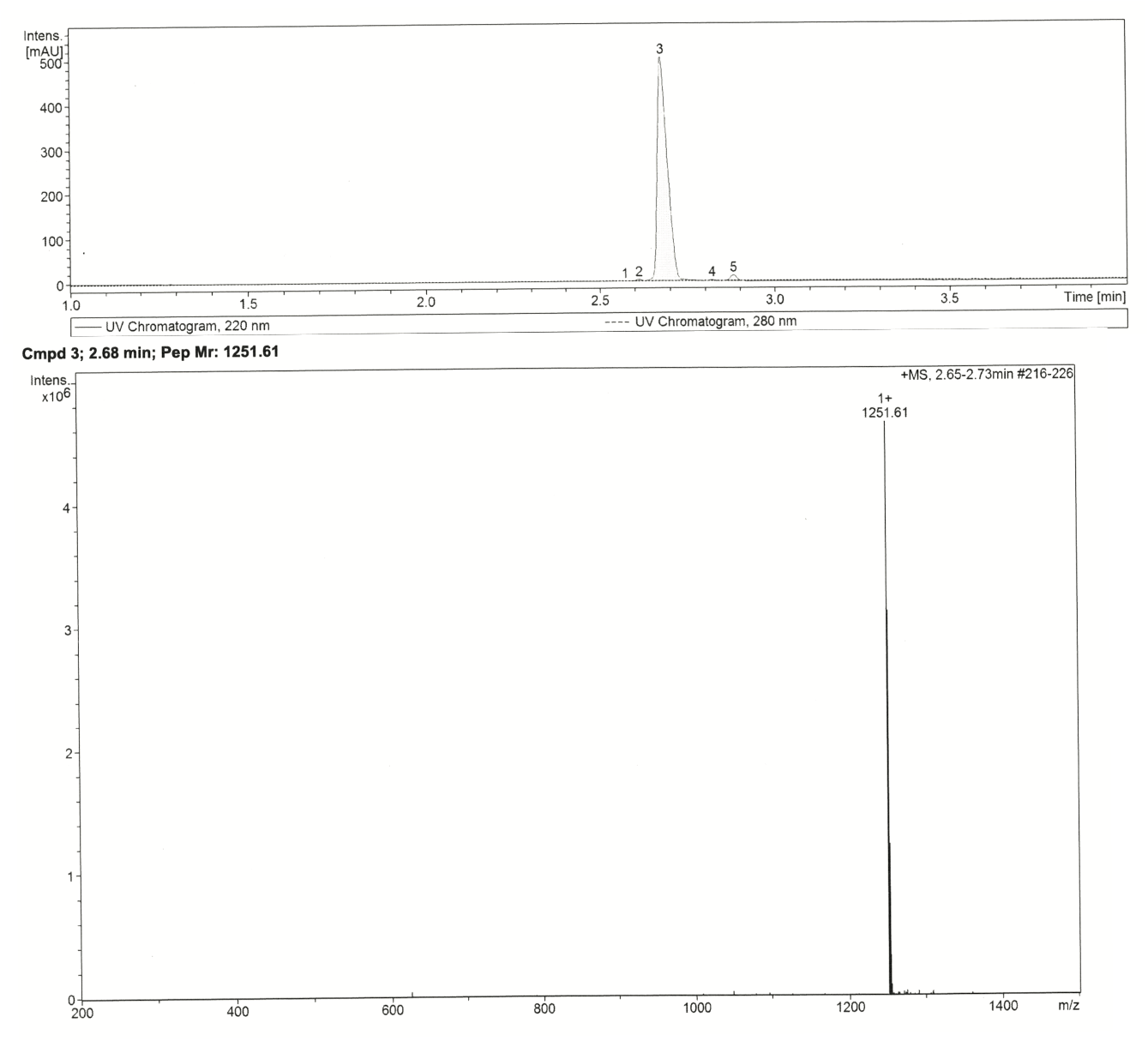
**

**Figure S7. Identity and purity of P450 and *b*_5_ binding region peptides.** Analysis by New England Peptides (supplier); *A*_220_ HPLC chromatograms and mass spectra.

**
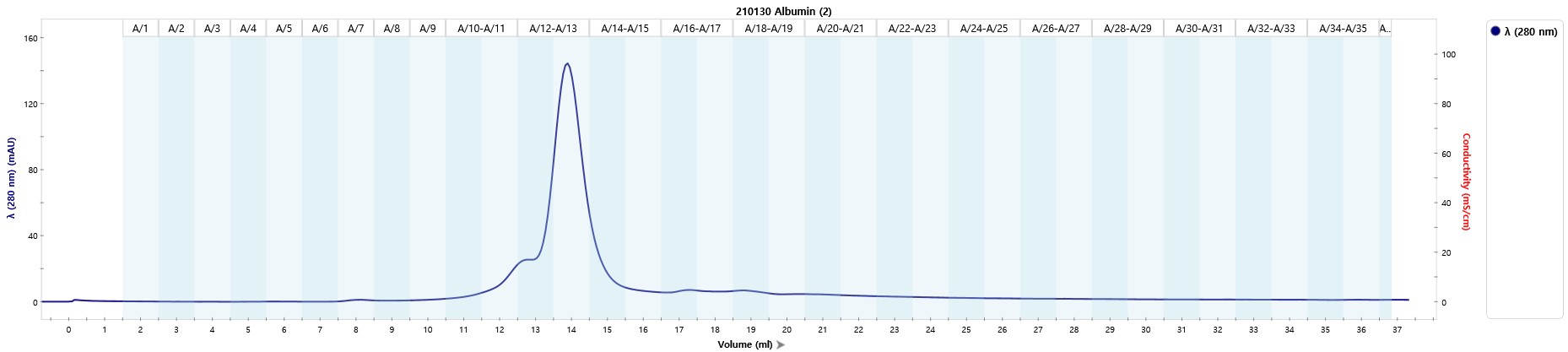

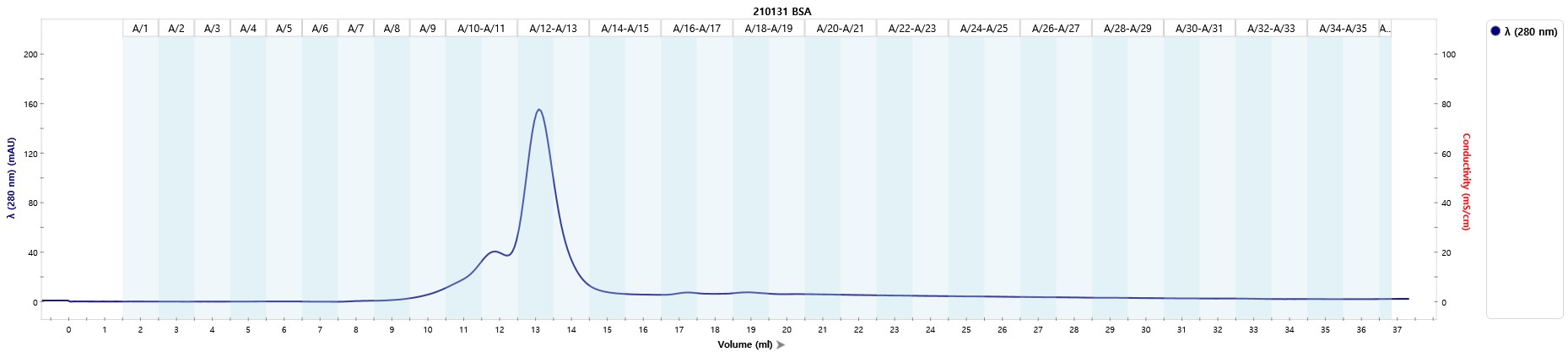

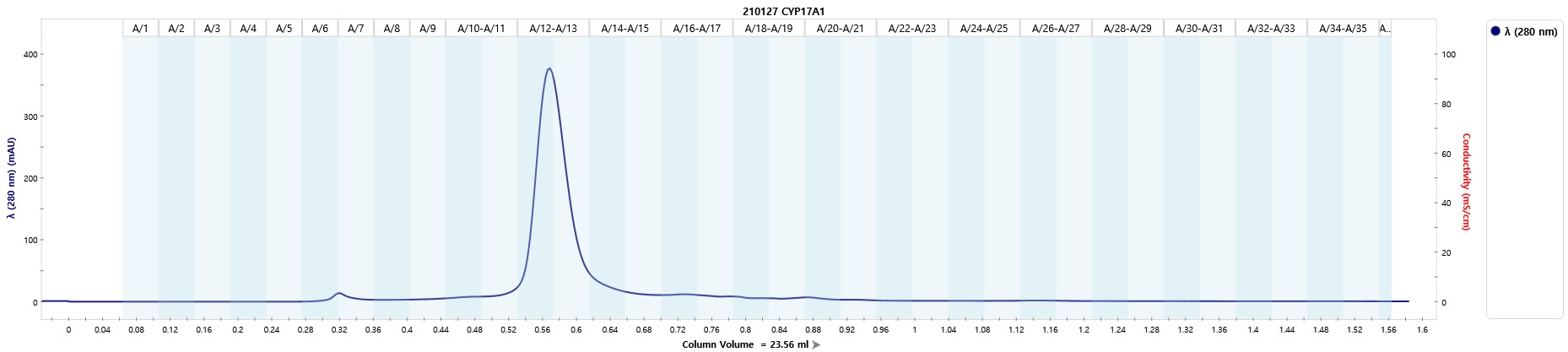
**

[17A1]

[OVA]

[BSA]

**Figure S8. Elution of P450 17A1 and ovalbumin and BSA standards from a Superose-12 gel filtration column.** Ovalbumin (OVA, 45 kDa), (P450) 17A1, BSA (monomer, 67 kDa). Absorbance was monitored at 280 nm.
